# Hypermethylation of lncRNA MEG3 impairs chemosensitivity of breast cancer cells

**DOI:** 10.1002/jcla.23369

**Published:** 2020-07-03

**Authors:** Hongchang Li, Puhua Wang, Jiazhe Liu, Weiyan Liu, Xubo Wu, Junbin Ding, Jie Kang, Jindong Li, Jingfeng Lu, Gaofeng Pan

**Affiliations:** ^1^ Department of General Surgery Institute of Fudan‐Minhang Academic Health System Minhang Hospital Fudan University Shanghai China

**Keywords:** breast cancer, chemoresistance, lncRNA MEG3, methylation

## Abstract

**Background:**

Chemoresistance posed a barrier to successful treatment of breast cancer (BC), and lncRNA MEG3 has been documented to implicate in BC development. However, whether MEG3 methylation, which led to low MEG3 expression, was relevant to BC progression and chemoresistance remained uncertain.

**Methods:**

In the aggregate, 374 pairs of tumor tissues and adjacent normal tissues were collected from pathologically confirmed BC patients, and four BC cell lines, including MDA‐MB‐231, Bcap‐37, MCF‐7, and SK‐BR‐3, were purchased. Moreover, methylation‐specific polymerase chain reaction (PCR) was adopted to evaluate the methylation status of BC tissues and cell lines, and chemo‐tolerance of BC cell lines was assessed by performing MTT assay. Concurrently, transwell assay and scratch assay were carried out to estimate the migratory and invasive capability of BC cell lines.

**Results:**

Methylated MEG3, lowly expressed MEG3, large tumor size (≥2 cm), advanced TNM grade and lymphatic metastasis were potentially symbolic of poor prognosis among BC patients (*P* < .05). Besides, MDA‐MB‐231 cell line exhibited the strongest resistance against paclitaxel, adriamycin, and vinorelbine (*P* < .05), while MCF‐7 cell line seemed more sensitive against these drugs than any other BC cell line (*P* < .05). Furthermore, pcDNA3.1‐MEG3 and 5‐Aza‐dC markedly sensitized MDA‐MB‐231 and MCF‐7 cell lines against the drug treatments (*P* < .05). Simultaneously, proliferation and metastasis of the BC cell lines were slowed down under the force of pcDNA3.1‐MEG3 and 5‐Aza‐dC (*P* < .05).

**Conclusion:**

Preventing methylation of MEG3 might matter in lessening BC chemoresistance, owing to its hindering proliferation and metastasis of BC cells.

## 1 INTRODUCTION

Breast cancer (BC), one common malignancy among global women, was usually sparked by abnormalities in breast epithelium. According to the statistics publicized by International Agency for Research on Cancer (IARC) in 2008, BC incidence has reached nearly 22.9% among worldwide females.^1^ Despite standard treatments established by National Comprehensive Cancer Network (NCCN) guideline, annually there were 20%‐40% of BC patients who suffered from BC recurrence and metastasis,^2^ and the 5‐year overall survival metastatic ones hovered around 25%.^3^ Hence, novel therapeutic means for BC were in desperate need, given the unoptimistic outcome of BC patients.

Breast cancer onset, a cumulative process, was correlative with aberrant activation of oncogenes and inactivation of anti‐oncogenes, which argued the significance of elucidating biomarkers that mirrored progression of BC. Long non‐coding RNAs (lncRNAs), a class of non‐coding RNAs longer than 200 nt, were acknowledged to modify the expression of protein‐coding genes in considerable aspects, and their abnormal expression was sometimes a reflection of cancer development and chemoresistance.^4^ For instance, highly expressed lncRNA HOX transcript antisense RNA (HOTAIR) and lncRNA H19 were found to quicken neoplastic growth and to facilitate tumor transfer.^5^, ^6^ Moreover, doxorubicin/etoposide tolerance of squamous cancer cells was intensified when lncRNA cancer up‐regulated drug resistant (CUDR) was highly expressed, which blocked apoptosis of tumor cells.^7^ Concerning lncRNA matriarchal‐expressed gene 3 (MEG3) studied here, its expression was down‐regulated and even lost in tumor tissues of brain, bladder, bone marrow, breast, cervix, colon, liver, lung, and prostate,^8^, ^9^, ^10^, ^11^, ^12^ as compared with normal tissues of these organs.^13^ In addition, over‐expressed MEG3, as disclosed by in vitro experiments, could impede BC proliferation and metastasis by enhancing transcription of p53‐targeting genes (eg, p21, Maspin, and KA11).^14^ The MEG3 was also able to reverse cisplatin‐tolerance of lung adenocarcinoma cells by inducing cell apoptosis,^15^ and silencing of MEG3, conversely, improved cisplatin‐resistance of non‐small cell lung cancer cells via activation of WNT/β‐catenin signaling.^16^ Based on the above, there presented a probability that expressional loss of MEG3 might advance neoplastic (eg, BC) progression and boost chemoresistance of malignancies.

It was noteworthy that hypermethylation of MEG3 promoter was a principal cause of lowly expressed MEG3 in various disorders (eg, BC).^17^, ^18^ To be specific, the methylation rate of MEG3 promotor was higher in pituitary tumor than in normal pituitary gland.^19^ And hypermethylation of MEG3 promotor was also discoverable among patients who were tortured by neurofibroma, meningioma, myelodysplastic syndrome, acute myelogenous leukemia, and acute myeloid leukemia.^10^, ^20^, ^21^, ^22^ It was, hence, conjectured that MEG3 methylation might also account for the role of lowly expressed MEG3 in regulating tumor activity, such as metastasis and chemoresistance. Nonetheless, up to now a limited number of investigations have been undertaken to verify this point. In response, clinical and in vitro experiments were designed here to overcome this handicap.

## 2 MATERIALS AND METHODS

### 2.1 Acquisition of clinical specimens

From March 2008 to January 2014, 374 pairs of tumor tissues and adjacent normal tissues were collected from pathologically confirmed BC patients, who received treatments in the medical oncology of Minhang Hospital. The BC patients were graded according to severity, based on the staging standard revised by World Health Organization (WHO) in 2003.^23^ The participants all complied with following items: (a) their diagnostic results agreed with criteria detailed in Chinese Anti‐Cancer Association Guidelines and Specifications on Breast Cancer Diagnosis and Treatment^24^; (b) they hardly underwent any treatments before; (c) they were able to communicate with medical staff smoothly; and (d) they have signed informed consents. Additionally, the subjects were eliminated from this project if they: (a) showed disorders in bilateral breasts, liver, and kidney, or (b) were complicated by other malignancies. Last but not the least, procedures of this project have obtained approvals from Minhang Hospital and its affiliated ethics committee.

### 2.2 Cell culture and cell transfection

MCF‐10A cell line and 4 BC cell lines (ie, MDA‐MB‐231 [ER−, PR−, HER2−], Bcap‐37 [ER−, PR−, HER2−], MCF‐7 [ER+, PR+, HER2−], and SK‐BR‐3 [ER−, PR−, HER2+]), purchased from American Type Culture Collection (ATCC), were cultivated in DMEM medium (Hyclone) which was blended by 10% fetal bovine serum (FBS), 100 U/mL penicillin, and 100 μg/mL streptomycin (Gibco).

Regarding cell transfection, pcDNA3.1‐MEG3 plasmid was constructed by sub‐cloning MEG3 sequences into pcDNA3.1 vector (GenePharma). When BC cell lines seeded at the density of 5 × 10^5^/well reached 70% confluence, pcDNA3.1‐MEG3, si‐MEG3‐1 (sense: 5′‐GCUCAUACUUUGACUCUAUTT‐3′, antisense: 5′‐AUAGAGUCAAAGUAUGAGCTT‐3′, GenePharma), and si‐MEG3‐2 (sense: 5′‐CCCUCUUGCUUGUCUUACUTT‐3′, antisense: 5′‐AGUAAGACAAGCAAGAGGGTT‐3′, GenePharma) were transfected into MDA‐MB‐231 and MCF‐7 cell lines with the help of Lipo‐fectamine 2000 reagent kit (Invitrogen). The BC cells after 48‐hour transfection were prepared to conduct real‐time PCR, and those having experienced 72‐hour transfection were collected to perform Western blotting.

### 2.3 Reverse‐transcription polymerase chain reaction

Breast Cancer tissues and cell lines were firstly lysed after supplementation of TRIzol reagent (Invitrogen), so as to extract total RNAs. Quality of the RNAs was guaranteed via UV spectrophotometer, and then, the RNAs were reversely transcribed into cDNAs (Invitrogen). With cDNAs as the template, real‐time PCR was launched as per specifications of Power SYBR Green kit (TaKaRa), and the reaction condition was designed as (a) pre‐denaturation at 95°C for 30 seconds, and (b) 40 cycles of denaturation at 95°C for 5 seconds, annealing at 60°C for 30 seconds, and extension at 72°C for 30 seconds. Finally, expression of MEG3 (upstream primer: 5′‐CTGCCCATCTACACCTCACG‐3′, downstream primer: 5′‐CTCTCCGCCGTCT‐GCGCTAGGGGCT‐3′) as relative to GAPDH (upstream primer: 5′‐GTCAACGGATTTGGTCTGTATT‐3′, downstream primer: 5′‐AGTCTTCTGGGTGGCAGTGAT‐3′) was calculated, after being normalized based on 2^−ΔΔ^ *^C^* ^T^ method.

### 2.4 Methylation‐specific PCR

Total DNAs were extracted from BC tissues and cell lines with aid of QIAmp DNA Mini kit (Qiagen), and their quality was assessed on a nucleic acid protein analyzer (model: BioPhotometer plus). The DNAs were uncontaminated by RNAs or proteins, when their OD260/OD280 ratio lied between 1.7 and 1.9. Subsequently, bisulfite modification of the DNAs was performed on a MyCycler PCR instrument, aided by EpiTect Bisulfite kit (Qiagen). With the help of MEG3 methylation primers (forward: 5′‐GTTAGTAATCGGGTTTGTCGGC‐3′, reverse: 5′‐AATCATAACTCCGAACACCCGCG‐3′) and un‐methylated primers (forward: 5′‐GAGGATGGTTAGTTATTGGGGT‐3′, reverse: 5′‐CCACCATAACCAACACCCTATAATCACA‐3′), Methylation‐specific PCR (MSP) was accomplished by consulting EpiTect MSP kit (Qiagen). The PCR products were visualized on the 2.5% agarose gel and then photographed by the gel imager (model: GDS 7500; UVP). Gray values of the strip images were quantitated utilizing Image J analysis software, and the experiments were repeated for 3 times.

### 2.5 Western blotting

Breast Cancer cell lines were treated with RIPA lysate (Beyotime), and the products were centrifuged to gather supernatants. The concentration of total proteins was monitored by relying on BCA kit (Beyotime). After denaturation at 100°C for 5 minutes, 30 μg protein of each sample was separated by feat of sodium dodecyl sulfate polyacrylamide gel electrophoresis (SDS‐PAGE), and then, gel of target proteins was electrically transferred onto polyvinylidene fluoride (PVDF) membrane. After blockage within 5% skimmed milk powder, the PVDF membrane was gently shaken at room temperature for 2 hours. Subsequently, the samples were incubated by rabbit anti‐human primary antibodies (Abcam) against E‐cadherin (1:500, ab15148), β‐catenin (1:5000, ab32572), N‐cadherin (1:1000, ab76057), and Vimentin (1:1000, ab92547) at 4°C for overnight, which was followed by incubation with goat anti‐rabbit IgG H&L (HRP) (1:2000, ab205718; Abcam) for another 1 hour. At last, development was carried through by means of enhanced chemi‐luminescence (ECL).

### 2.6 Cell apoptosis assay

The digested BC cells were centrifuged at 1000 *g*, and then, binding buffer was added to re‐suspend the cells. After that, cell suspension, mixed by Annexin V‐FITC (Invitrogen), was placed quiescently in the dark for 10 minutes. Then, the BC cells were stained by propidium iodide (PI) dye solution at room temperature for another 10 minutes. Apoptotic rate of the BC cells was measured utilizing flow cytometry (Thermo Fisher), with an excitation (Ex) wavelength of 448 nm and an emission wavelength (Em) of 530 nm.

### 2.7 MTT assay for assessing chemosensitivity of BC cells

Breast Cancer cells seeded at the concentration of 5 × 10^3^/well were handled by gradient concentrations of 5‐fluorouracil (5, 10, 15, 25, 50, and 100 μg/mL) (Sigma Chemical), paclitaxel (0.10, 1.00, 10, 25, and 50 μg/mL) (Sigma Chemical), adriamycin (0.25, 0.50, 1.00, 2, and 4 μg/mL) (Sigma Chemical), and vinorelbine (5, 10, 20, 40, and 60 μg/mL) (Pierre Fabre Pharma, France) for 48 hours. Precisely, 5 μL MTT solution (concentration: 5 g/L) was supplemented into each well, and the cells were then cultivated at 37°C for 4 hours. After careful removal of the nutrient solution, 150 μL DMSO was dropped into each well, until sediments were completely dissolved. The absorbance value (OD value) of each well was measured using a microplate reader (Thermo) at 490 nm, and the inhibition rate of cell growth (%) was calculated in line with the formula of [1 − OD_experiment_/OD_control_] × 100%. Besides, the half inhibitory concentration of drugs (IC50) was calculated from the linear regression curve produced by different concentrations of drugs and corresponding cell growth inhibition rate.^25^

### 2.8 Transwell invasion assay

On one hand, 100 μL matrigel, diluted by serum‐free and high‐glucose DMEM at a ratio of 1:8, was added into each Transwell chamber, and the gel was incubated at 37°C until solidification. Then, 1 × 10^5^ BC cells were added onto the Matrigel of each well. On the other hand, 10% FBS‐containing DMEM (700 μL) was added to the lower transwell chamber. After 24‐hour culture, 10% methanol was employed to fix the BC cells for 20 minutes, followed by staining the BC cells with crystal violet for 10 minutes. The cotton swab was adopted to wipe out cells which failed to penetrate the upper chamber. Eventually, cells taken from five random fields were counted under the light microscope at magnification × 200.

### 2.9 Cell scratch test

Well‐grown MDA‐MB‐231 and MCF‐7 cell lines, adjusted to a concentration of 2 × 10^5^/mL, were incubated into 12‐well plates. When the cells grew to about 80%‐90% confluence, a uniform line was drawn on the back of each well with a 10‐μL pipette tip. At the time points of 0 hour and 24 hours, photographs of the scratches were taken under the microscope, and Image J software was employed to measure the width of scratches.

### 2.10 Statistical analyses

All experimental data were analyzed using SPSS 13.0 statistical software. Comparisons between/among measurement data, expressed as mean ± standard deviation (SD), were accomplished by means of t test or one‐way analysis of variance (ANOVA), while the enumeration data were contrasted through usage of chi‐square test. The statistical difference was considered as significant in the context of *P* < .05.

## 3 RESULTS

### 3.1 MEG3 was lowly expressed and methylated in BC tissues and cell lines

**Figure 1:**
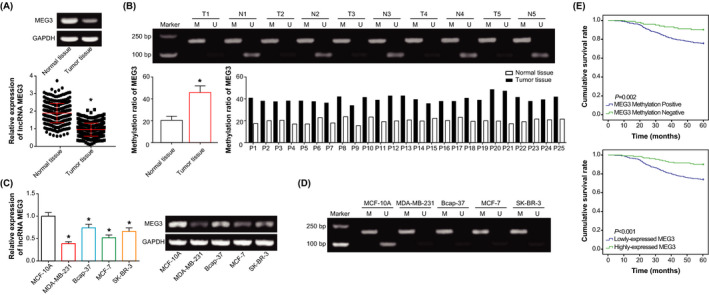
Clinical importance of MEG3 in forecasting prognosis of breast cancer patients. A, Comparison of lncRNA MEG3 expression between breast cancer tissues and para‐carcinoma tissues. *P < .05 when compared with para‐carcinoma tissues. B, Methylation rate of breast cancer tissues and para‐carcinoma tissues. *P < .05 when compared with para‐carcinoma tissues. C, LncRNA MEG3 expression within breast cancer cell lines (ie, MDA‐MB‐231, Bcap‐37, MCF‐7 and SK‐BR‐3) and human mammary epithelial cell line (ie, MCF‐10A). *P < .05 when compared with MCF‐10A. D, Methylated level of MEG3 in breast cancer cell lines (ie, MDA‐MB‐231, Bcap‐37, MCF‐7, and SK‐BR‐3) and human mammary epithelial cell line (ie, MCF‐10A). E, Association of under‐expressed MEG3 and methylated MEG3 with low survival of breast cancer patients

It was demonstrated that the expression level of MEG3 was obviously decreased in BC tissues when compared with adjacent normal tissues (*P* < .05) (Figure 1A). At the meantime, the methylation rate of MEG3 was significantly raised in BC tissues as compared with adjacent normal tissues (*P* < .05) (Figure 1B). Analogously, BC cell lines, including MDA‐MB‐231, Bcap‐37, MCF‐7, and SK‐BR‐3, were associated with lower MEG3 level and higher methylation rate of MEG3 than MCF‐10A cell line (*P* < .05) (Figure 1C,D).

The BC patients were further divided into methylation group (n = 272) and non‐methylation group (n = 102), depending on the methylation status of MEG3. Results of Table 1 elaborated that MEG3 methylation was associated with low MEG3 expression, large tumor size (≥2 cm), advanced TNM grade, and high histological grade (ie, G3) (all *P* < .05), rather than age, ER status, PR status, HER2 status, and lymphatic metastasis (*P* > .05), of BC patients. Besides, MEG3 methylation (HR: 2.14, 95% CI: 1.02‐4.51), low MEG3 expression (HR: 3.01, 95 CI%: 1.54‐5.87), large tumor size (≥ 2 cm) (HR: 1.92, 95 CI%: 1.12‐3.33), advanced TNM grade (HR: 1.79, 95%CI: 1.02‐3.13), and lymphatic metastasis (HR: 1.92, 95CI%: 1.11‐3.23) were likely to indicate poor prognosis of BC patients, after removing impacts exerted by other factors (Table 2). The Kaplan‐Meier analysis also vividly showed that BC patients who were featured by MEG3 methylation or low MEG3 expression were associated with shorter longevity than those with non‐methylated MEG3 or high MEG3 expression (*P* < .05) (Figure 1E).

**Table 1:** Correlation between MEG3 methylation status and clinical characteristics of breast cancer patients

Characteristics	MEG3 methylation				χ^2^	*P* value
	Positive		Negative			
MEG3 expression						
<3.46	189	69.49%	54	52.94%	8.92	.003
≥3.46	83	30.51%	48	47.06%		
Age						
<55 y	161	59.19%	67	65.69%	1.32	.252
≥55 y	111	40.81%	35	34.31%		
Tumor size						
<2 cm	164	60.29%	77	75.49%	7.48	.006
≥2 cm	108	39.71%	25	24.51%		
ER status						
Negative	209	76.84%	73	71.57%	1.11	.292
Positive	63	23.16%	29	28.43%		
PR status						
Negative	124	45.59%	42	41.18%	0.59	.444
Positive	148	54.41%	60	58.82%		
HER2 status						
Negative	137	50.37%	54	52.94%	0.20	.658
Positive	135	49.63%	48	47.06%		
TNM stage						
I/II	123	45.22%	60	58.82%	5.49	.019
III	149	54.78%	42	41.18%		
Histological grade						
G1/2	178	65.44%	79	77.45%	4.98	.026
G3	94	34.56%	23	22.55%		
Lymph node metastasis						
Negative	169	62.13%	74	72.55%	3.54	.060
Positive	103	37.87%	28	27.45%		

Abbreviations: ER, estrogen receptor; HER2, human epidermal growth factor receptor 2; PR, progesterone receptor.

**Table 2:** Clinical characteristics and breast cancer patients' overall survival

Characteristics	Univariate analysis			Multivariate analysis		
	Hazard ratio	95% CI	*P* value	Hazard ratio	95% CI	*P* value
MEG3 methylation						
Positive vs Negative	2.95	1.45‐5.99	.003	2.14	1.02‐4.51	.045
MEG3 expression						
<3.46 vs ≥3.46	3.18	1.67‐6.03	<.001	3.01	1.54‐5.87	.001
Age (y)						
<55 vs ≥55	1.05	0.62‐1.76	.860	1.16	0.67‐2.02	.591
Tumor size						
≥2 cm vs <2 cm	1.85	1.12‐3.13	.017	1.92	1.12‐3.33	.017
ER status						
Negative vs Positive	0.97	0.54‐1.74	.928	0.94	0.51‐1.76	.855
PR status						
Negative vs Positive	1.02	0.61‐1.69	.945	0.93	0.54‐1.60	.796
HER2 status						
Negative vs Positive	1.24	0.75‐2.05	.413	1.22	0.71‐2.10	.481
TNM stage						
III vs I/II	2.00	1.18‐3.33	.010	1.79	1.02‐3.13	.041
Histological grade						
G3 vs G1/2	1.18	0.69‐2.00	.538	1.01	0.57‐1.82	.960
Lymph node metastasis						
Positive vs Negative	1.92	1.15‐3.23	.012	1.92	1.11‐3.23	.019

Abbreviations: ER, estrogen receptor; HER2, human epidermal growth factor receptor 2; PR, progesterone receptor.

### 3.2 Inhibitory effect of chemotherapeutic drugs on growth of BC cell lines

**Figure 2:**
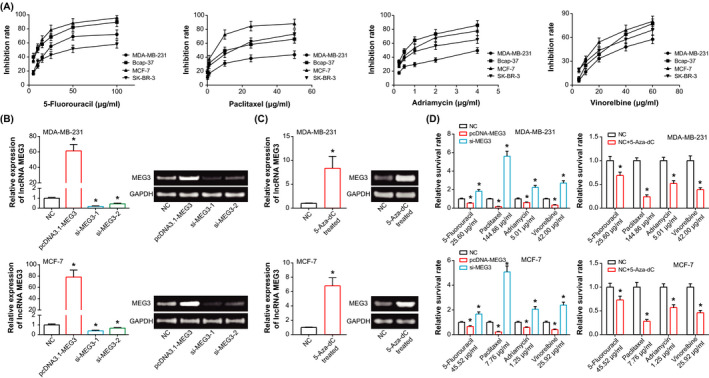
Sensitivity of breast cancer cell lines in response to 5‐fluorouracil, paclitaxel, adriamycin, and vinorelbine. A, Inhibition of 5‐fluorouracil, paclitaxel, adriamycin, and vinorelbine on the growth of breast cancer cell lines (ie, MDA‐MB‐231, Bcap‐37, MCF‐7, and SK‐BR‐3). B, MEG3 expression in MDA‐MB‐231 and MCF‐7 cell lines that were treated by NC, pcDNA3.1‐MEG3, si‐MEG3‐1, and si‐MEG3‐2. *P < .05 when compared with NC. C, MEG3 expression in MDA‐MB‐231 and MCF‐7 cell lines that were managed by 5‐Aza‐dC. *P < .05 when compared with NC. D, Survival rate of MDA‐MB‐231 and MCF‐7 cell lines under treatments of 5‐fluorouracil, paclitaxel, adriamycin, and vinorelbine in Nc, pcDNA3.1‐MEG3, si‐MEG3 group or NC, NC + 5‐Aza‐dC group. *P < .05 when compared with NC

As illustrated in Figure 2A, MDA‐MB‐231 cell line displayed the strongest tolerance against paclitaxel (IC50 = 144.86 μg/mL), adriamycin (IC50 = 5.01 μg/mL), and vinorelbine (IC50 = 42.00 μg/mL). MCF‐7 cell line was highly sensitive to treatments of 5‐fluorouracil (IC50 = 8.85 μg/mL), paclitaxel (IC50 = 1.49 μg/mL), and vinorelbine (IC50 = 18.13 μg/mL), as compared with other cell lines. Furthermore, growth of Bcap‐37 cell line was largely suppressed by adriamycin (*P* < .05), and SK‐BR‐3 cell line could resist against the harm brought by 5‐fluorouracil (*P* < .05). Based on the above, BC cells lines with strong chemo‐tolerance (ie, MDA‐MB‐231 cell line) and with high chemosensitivity (ie, MCF‐7 cell line) were selected for conducting following experiments.

### 3.3 Effects of MEG3 and 5‐Aza‐dC on chemo‐tolerance of BC cell lines

Whether in MDA‐MB‐231 cell line or in MCF‐7 cell line, pcDNA3.1‐MEG3 significantly elevated MEG3 expression to around 61.38‐fold to 78.25‐fold of control group (*P* < .05) (Figure 2B). By contrast, both si‐MEG3‐1 and si‐MEG3‐2 reduced MEG3 expression to merely 18%‐67% of control group (*P* < .05). As si‐MEG3‐1 led to more visible expressional reduction of MEG3 than si‐MEG3‐2 (*P* < .05), the si‐MEG3‐1 was utilized for subsequent cellular experiments. For another, MEG3 expression in MDA‐MB‐231 and MCF‐7 cell lines, which were treated by 5‐Aza‐dC, was increased to 6.81‐8.34 times of NC group (*P* < .05) (Figure 2C).

Moreover, transfection of pcDNA3.1‐MEG3 or treatment of 5‐Aza‐dC could markedly reduce the chemoresistance of MDA‐MB‐231 and MCF‐7 cell lines, when compared with NC group (*P* < .05) (Figure 2D). The survival rate of BC cells in the pcDNA3.1‐MEG3 group was greater than that of 5‐Aza‐dC‐treated BC cells (*P* < .05). Conversely, silencing of MEG3 resulted in stronger chemo‐tolerance of MDA‐MB‐231 and MCF‐7 cell lines in comparison with control group (*P* < .05) (Figure 2D).

### 3.4 Influence of MEG3 and 5‐Aza‐dC on viability, apoptosis, migration, and invasion of BC cell lines

**Figure 3:**
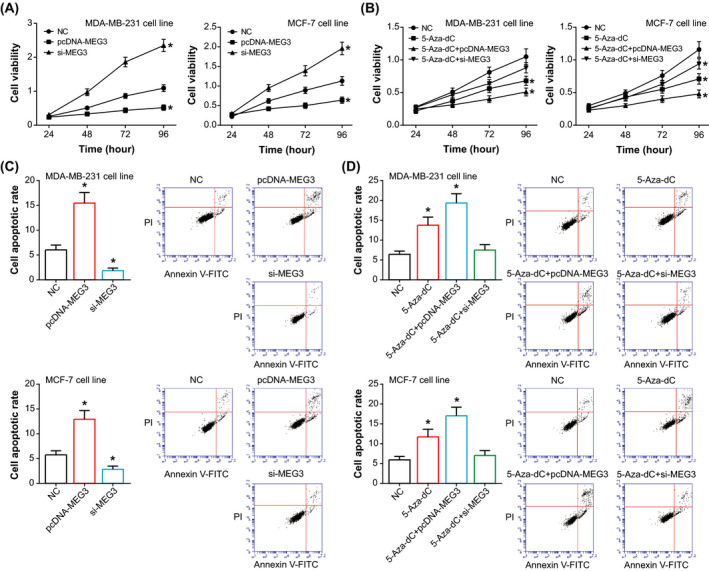
Contribution of MEG3 and 5‐Aza‐dC to viability and apoptosis of breast cancer cell lines. A, Viability of MDA‐MB‐231 and MCF‐7 cell lines that were transfected by pcDNA3.1‐MEG3 and si‐MEG3. *P < .05 when compared with NC. B, Viability of MDA‐MB‐231 and MCF‐7 cell lines that were handled by 5‐Aza‐dC, 5‐Aza‐dC + pcDNA3.1‐MEG3 and 5‐Aza‐dC + si‐MEG3. *P < .05 when compared with NC. C, Apoptosis of MDA‐MB‐231 and MCF‐7 cell lines that were transfected by pcDNA3.1‐MEG3 and si‐MEG3. *P < .05 when compared with NC. D, Apoptosis of MDA‐MB‐231 and MCF‐7 cell lines in the NC, 5‐Aza‐dC, 5‐Aza‐dC + pcDNA3.1‐MEG3, and 5‐Aza‐dC + si‐MEG3 groups. *P < .05 when compared with NC

Over‐expressed MEG3 was found to impair viability of MDA‐MB‐231 and MCF‐7 cell lines (*P* < .05), while lowly expressed MEG3 reinforced viability of the BC cells to 1.73‐2.16 times of control group (*P* < .05) (Figure 3A). Furthermore, 5‐Aza‐dC treatment decreased viability of BC cells less significantly than combined treatments of 5‐Aza‐dC and pcDNA3.1‐MEG3 (*P* < .05), and si‐MEG3 could counteract the effects of 5‐Aza‐dC on viability of BC cells (*P* < .05) (Figure 3B). Contrary to the trend of cell viability, the apoptotic rate of MDA‐MB‐231 and MCF‐7 cell lines was heightened evidently in the pcDNA3.1‐MEG3 group (*P* < .05), while si‐MEG3 inhibited apoptosis of the cell lines significantly in comparison with NC group (*P* < .05) (Figure 3C). Moreover, the apoptotic rate of 5‐Aza‐dC + pcDNA‐MEG3 group was above that of 5‐Aza‐dC group (*P* < .05), yet 5‐Aza‐dC + si‐MEG3 group resulted in a significant decrease of apoptotic rate as compared with 5‐Aza‐dC group (*P* < .05) (Figure 3D).

**Figure 4:**
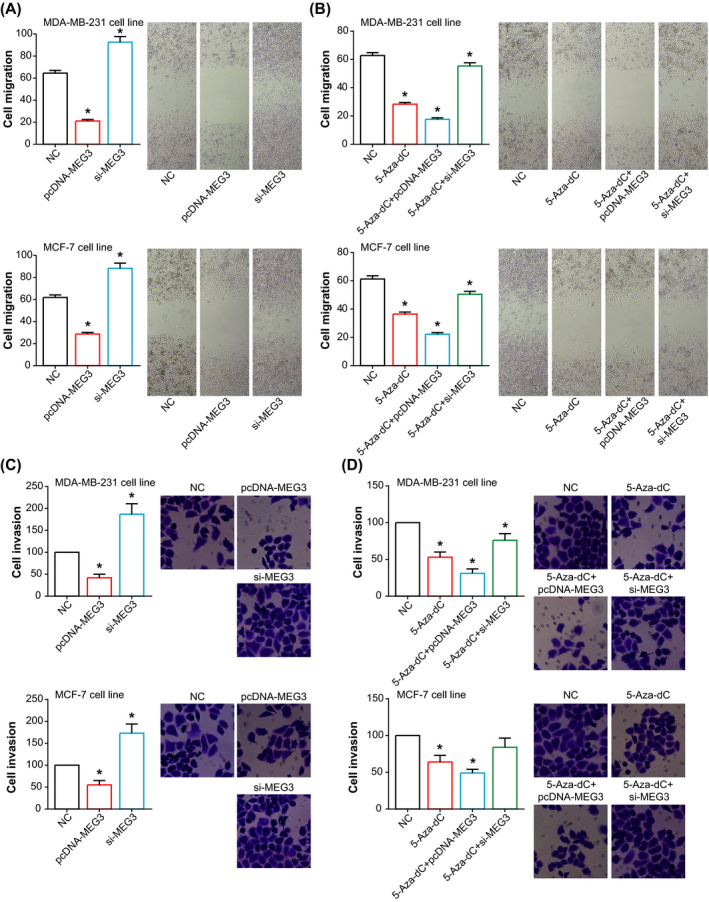
Role of MEG3 and 5‐Aza‐dC in regulating migration and invasion of breast cancer cell lines. A, Migratory potency of MDA‐MB‐231 and MCF‐7 cell lines in the NC, pcDNA3.1‐MEG3, and si‐MEG3 groups. *P < .05 when compared with NC. B, Migratory capability of MDA‐MB‐231 and MCF‐7 cell lines that were managed through approaches of NC, 5‐Aza‐dC, 5‐Aza‐dC + pcDNA3.1‐MEG3, and 5‐Aza‐dC + si‐MEG3. *P < .05 when compared with NC. C, Invasive trend of MDA‐MB‐231 and MCF‐7 cell lines that were transfected by pcDNA3.1‐MEG3 and si‐MEG3. *P < .05 when compared with NC. D, Invasive potency of MDA‐MB‐231 and MCF‐7 cell lines that were treated through ways of NC, 5‐Aza‐dC, 5‐Aza‐dC + pcDNA3.1‐MEG3, and 5‐Aza‐dC + si‐MEG3. *P < .05 when compared with NC

**Figure 5:**
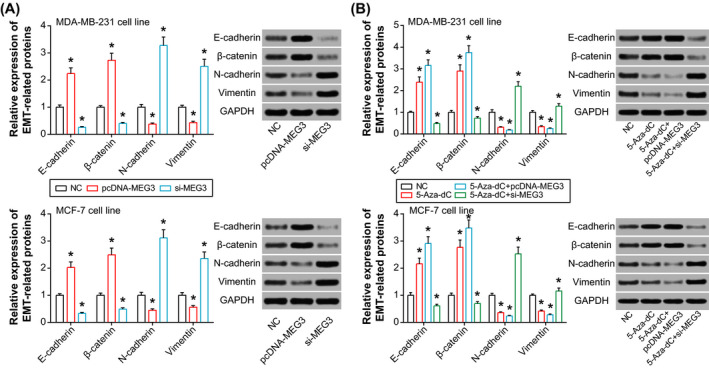
Effects of MEG3 and 5‐Aza‐dC on expression of EMT‐related proteins within breast cancer cell lines. A, Determination of EMT‐related proteins within MDA‐MB‐231 and MCF‐7 cell lines that were transfected by pcDNA3.1‐MEG3 and si‐MEG3. *P < .05 when compared with NC. B, Examination of EMT‐specific proteins within MDA‐MB‐231 and MCF‐7 cell lines that were treated by 5‐Aza‐dC, 5‐Aza‐d + pcDNA3.1‐MEG3, and 5‐Aza‐d + si‐MEG3. *P < .05 when compared with NC

Furthermore, both migration and invasion of MDA‐MB‐231 and MCF‐7 cell lines were obviously promoted after silencing of MEG3 (*P* < .05), whereas transfection of pcDNA3.1‐MEG3 engendered an opposite tendency of the indicators (*P* < .05) (Figure 4A,C). Besides, 5‐Aza‐dC in combination with pcDNA3.1‐MEG3 was outstanding in inhibiting migration and invasion of BC cells, when compared with NC group (*P* < .05) (Figure 4B,D). And 5‐Aza‐dC combined with si‐MEG3 facilitated migration and invasion of MDA‐MB‐231 and MCF‐7 cell lines more strongly than 5‐Aza‐dC alone (*P* < .05) (Figure 4B,D). Concerning EMT‐related proteins, MEG3 was observed to encourage E‐cadherin/β‐catenin expression and yet depress N‐cadherin/Vimentin expression in both MDA‐MB‐231 and MCF‐7 cell lines (*P* < .05) (Figure 5A). Treatment of 5‐Aza‐dc also impeded N‐cadherin/Vimentin expression and promoted E‐cadherin/β‐catenin expression within BC cells, when compared with NC group (*P* < .05) (Figure 5B).

## 4 DISCUSSION

LncRNAs, characterized by specific secondary structure and highly conserved local sequence, were no longer neglected regarding their tight linkage with either development or drug resistance of neoplasms.^26^, ^27^, ^28^ In particular, lncRNAs BCAR4,^29^ GAS5,^30^ HOTAIR,^31^ HIF1A‐AS2, AK124454,^32^, ^33^ ATB,^34^ and UCA1^35^ were involved in inducing drug‐tolerance of various cancer cells by acting upon respective downstream molecules. As for BC, lncRNA H19 was implied to promote BC metastasis,^36^ while lncRNA HOTAIR amplified BC risk via deactivation of JAM2 and PCDH.^6^ Despite these advances, the biological network that connected lncRNAs with chemotherapeutic efficacy of BC remained far from complete.

As a supplement, lncRNA MEG3 was introduced in this investigation, considering its governing neoplastic chemosensitivity through intricate regulation of cell activity. For instance, over‐expression of MEG3 was capable of diminishing transduction of AKT signaling, which finally restrained growth and invasion of BC cells and also put a brake on BC angiogenesis.^37^ Besides, reducing MEG3 expression, engendered by dysfunctional pRb‐DNMT1 signaling, was documented to boost multiplication of lung cancer cells.^38^ Furthermore, MEG3 hindered endometrial cancer development by deactivation of Notch signaling,^39^ and it could suppress epithelial mesenchymal transition (EMT) of BC cells by negative regulation of miR‐421.^40^ Our investigation also revealed that MEG3 served as an effective restraint of BC metastasis and proliferation (Figures 1, 2, 3, 4, 5). Of note, BC cells of distinct subtypes were investigated here, which showed that MEG3 expression was the lowest in MDA‐MB‐231 cell line (ER‐, PR‐, HER2‐). However, MEG3 expression seemed statistically irrelevant to ER, PR or HER2 status of the recruited BC patients (Tables 1 and 2). In future a large number of BC patients had better be recruited to confirm this result, since that a limited sample size (Table 1) might blur the inherent association of MEG3 with BC subtypes.

In addition, EMT was increasingly certified as an accompanying phenomenon when tumor cells exhibited drug‐resistant inclinations.^41^ For instance, epithelial‐derived malignant cells not only expressed biomarkers that were indicative of mesenchymal differentiation, but also became less sensitive to chemo‐treatments.^42^, ^43^, ^44^ Hence, the incremental chemosensitivity of BC cells might, to some extent, result from the inhibition of MEG3 on EMT process of cancer cells (Figure 5). Besides EMT, drug resistance of cancer cells could also be facilitated by restraining apoptosis of tumor cells, and lncRNAs appeared as crucial biomarkers in modulating apoptosis of neoplastic cells. For instance, knockout of lnc‐AK022798 in cisplatin‐tolerant gastric cancer cells was found to heighten Caspase‐3/8 expression and to propel apoptosis of the tumor cells.^45^ Here, over‐expressed MEG3 (ie, pcDNA‐MEG3 transfection) and de‐methylated MEG3 (ie, 5‐Aza‐dC treatment) were found to elevate the apoptotic rate of MDA‐MB‐231 and MCF‐7 cell lines (Figure 3C,D), which suggested that enhancive BC apoptosis might be another mechanism explaining MEG3’ reinforcing BC chemosensitivity. However, here we failed to figure out downstream molecules that aided MEG3 to play such roles, which awaited further researches.

Furthermore, epigenetic silencing of tumor‐suppressive genes was, to some degree, insinuated to result from super‐methylation of CpG island (CGI) in the promoter of these genes. Taking MEG3 for instance, its promoter was abundant with CGI which carried IG‐DMR and MEG3‐DMR, and methylation of its promoter could trigger expressional loss of MEG3 in tumors.^46^ Apart from that, clinical evidence also demonstrated that the methylation frequency of MEG3 in NSCLC tissues (ie, 96%) exceeded that in normal tissues (ie, 68%), and MEG3 expression was lower in NSCLC tissues than in normal tissues.^47^ Results of in vitro studies also pointed out that lowly expressed MEG3 in ovarian cancer cells was relevant to hypermethylation in the promoter of MEG.^48^ Consistently, the methylation rate of MEG3 in BC tissues was also obviously higher than that in adjacent non‐tumor tissues (Figure 1B). What's more, BC cell lines were methylated as relative to normal cell line (Figure 1D), and de‐methylation of MEG3 was capable of accelerating metastasis and enhancing chemosensitivity of BC cell lines (Figure 2D). All these indicated that MEG3 methylation might be responsible for the role of under‐expressed MEG3 in boosting EMT and chemo‐tolerance of BC, yet more detailed proofs were in demand.

Conclusively, down‐regulated MEG3 expression, partly on account of hypermethylation of MEG3 promotor, could be a mighty pusher for BC progression, and targeting MEG3 might be a promising manner to relieve BC chemoresistance. Nevertheless, a series of shortcomings were present within this investigation. Firstly, we failed to group BC patients according to their status of estrogen receptor (ER), progesterone receptor (PR), or human epidermal growth receptor 2 (HER2), so it was tough to associate MEG3 with BC of distinct subtypes. Secondly, the manner through which MEG3 was methylated in BC tissues and cell lines was not elucidated here. Thirdly, animal models were not established to verify whether methylated MEG3 might depress tumor growth. Above all, all these questions raised were in need of settlements.
